# Activation of the Calcium-Sensing Receptor Corrects the Impaired Mitochondrial Energy Status Observed in Renal Polycystin-1 Knockdown Cells Modeling Autosomal Dominant Polycystic Kidney Disease

**DOI:** 10.3389/fmolb.2018.00077

**Published:** 2018-08-24

**Authors:** Annarita Di Mise, Marianna Ranieri, Mariangela Centrone, Maria Venneri, Grazia Tamma, Daniela Valenti, Giovanna Valenti

**Affiliations:** ^1^Department of Biosciences, Biotechnologies and Biopharmaceutics, University of Bari, Bari, Italy; ^2^Istituto Nazionale di Biostrutture e Biosistemi, Rome, Italy; ^3^Institute of Biomembranes Bioenergetics and Molecular Biotechnologies, National Council of Research, Bari, Italy; ^4^Center of Excellence in Comparative Genomics, University of Bari, Bari, Italy

**Keywords:** calcium-sensing receptor, renal channelopathies, calcimimetics, ciPTEC, mitochondria, ATP

## Abstract

Autosomal Dominant Polycistic kidney Disease (ADPKD) is a renal channelopathy due to loss-of-function mutations in the *PKD1* or *PKD2* genes, encoding polycystin-1 (PC1) or polycystin-2 (PC2), respectively. PC1 is a large protein found predominantly on the plasma membrane where interacts with different proteins, including PC2. PC2 is a smaller integral membrane protein also expressed in intracellular organelles, acting as a non-selective cation channel permeable to calcium. Both PC1 and PC2 are also localized to the primary cilium of renal epithelial cells serving as mechanosensor that controls calcium influx through the plasma membrane and regulates intracellular calcium release from the endoplasmic reticulum. The mechanisms by which PC1/2 dysfunction leads to ADPKD needs still to be clarified. We have recently reported that selective Calcium-Sensing Receptor (CaSR) activation in human conditionally immortalized Proximal Tubular Epithelial cells deficient for PC1 (ciPTEC-PC1KD), deriving from urine sediments reduces intracellular cAMP and mTOR activity, and increases intracellular calcium reversing the principal ADPKD dysregulations. Reduced cellular free calcium found in ADPKD can, on the other hand, affect mitochondrial function and ATP production and, interestingly, a relationship between mitochondria and renal polycystic diseases have been suggested. By using ciPTEC-PC1KD as experimental tool modeling of ADPKD, we show here that, compared with wild type cells, ciPTEC-PC1KD have significantly lower mitochondrial calcium levels associated with a severe deficit in mitochondrial ATP production, secondary to a multilevel impairment of oxidative phosphorylation. Notably, selective CaSR activation with the calcimimetic NPS-R568 increases mitochondrial calcium content close to the levels found in resting wild type cells, and fully recovers the cell energy deficit associated to the PC1 channel disruption. Treatment of ciPTEC-PC1KD with 2-APB, an IP3R inhibitor, prevented the rescue of bioenergetics deficit induced by CaSR activation supporting a critical role of IP3Rs in driving ER-to-mitochondria Ca2+ shuttle. Together these data indicate that, besides reversing the principal dysregulations considered the most proximal events in ADPKD pathogenesis, selective CaSR activation in PKD1 deficient cells restores altered mitochondrial function that, in ADPKD, is known to facilitate cyst formation. These findings identify CaSR as a potential therapeutic target.

## Introduction

Loss-of-function of polycystin-1 (PC1) or polycystin-2 (PC2), two trans-membrane proteins that form a heteromeric molecular complex in the cilia and plasma membrane, results in the most common life-threatening genetic renal disorder, called Autosomal Dominant Polycystic Kidney Disease (ADPKD), characterized by the formation and development of kidney cysts (Torres et al., 2007). Biochemical experiments have shown that PC1 and PC2 form a receptor-ion channel complex (Hanaoka et al., 2000; Yu et al., 2009) leading ADPKD to be classified among channelopathies, a heterogeneous group of disorders caused by the dysfunction of ion channels expressed in cellular membranes and many intracellular organelles.

ADPKD affects 1:400–1:1,000 worldwide and 50% of PKD patients will require dialysis or kidney transplantation within 60 years of age (Harris and Torres, 2014). It results from mutations in *PKD1* or *PKD2* genes (encoding PC1 or PC2, respectively) (Hughes et al., 1995; Mochizuki et al., 1996) which cause analogous clinical signs. The close identity of the disease manifestations, independently of the responsible gene, suggests that PC1 and PC2 are involved in a common signaling pathway.

PC1 is considered a mechanosensor receptor with a large extracellular N-terminal, 11 transmembrane domains and a cytoplasmic C-terminal region (Hughes et al., 1995; Oatley et al., 2012). The N-terminal domain contains several structural motifs found to be essential for interactions with extracellular proteins and carbohydrates (Löhning et al., 1996). The C-terminal cytoplasmic domain contains a coiled-coil sequence that binds specifically to the C-terminus of PC2 (Qian et al., 1997), and it has been shown that, once cleaved from the full length PC1, it translocates to the nucleus (Chauvet et al., 2004; Chapin et al., 2010). Interestingly, PC2 expression is able to attenuate PC1 C-terminal tail translocation, modulating its signaling properties (Chauvet et al., 2004).

PC2, also known as transient receptor potential cation channel, subfamily P, member 2 (TRPP2), is a nonselective cation channel containing 6 transmembrane domains, with both N- and C-terminal tails facing the cytoplasm (Mochizuki et al., 1996; Celić et al., 2008). The N-terminal portion carries a ciliary targeting domain (Geng et al., 2006), while the C-terminal tail includes an EF-hand, and a coiled coil motif whose binding to the coiled-coil domain in the PC1 C terminus is critical for the formation of the PC1-PC2 complex (Yu et al., 2009). Conversely, other experiments showed that PC1 and PC2 interaction is preserved in systems not expressing the coiled-coil domain and is dependent on the N-terminal domain (Babich et al., 2004; Feng et al., 2008; Celić et al., 2012). However, the factors that regulate the localization, trafficking, and channel activity of the polycystins remain still unclear.

The polycystins form a complex that localizes to the primary cilium, where it may be involved in chemosensory or mechanosensory pathways (Pazour and Rosenbaum, 2002; Chapin and Caplan, 2010). In addition, the PC complex is expressed on the plasma membrane, as well as in intracellular compartments, where PC2 can regulate Ca^2+^ release from the endoplasmic reticulum (Koulen et al., 2002). Many studies propose that PC1 and PC2 may reciprocally affect each other's membrane or ciliary localization (Harris et al., 1995; Ong and Wheatley, 2003; Xu et al., 2007), and importantly, the interaction between the polycystins has been suggested to be crucial in defining the properties of the ion channel associated with the complex (Hanaoka et al., 2000; Delmas et al., 2004).

In ADPKD, disruption of the PC complex, resulting from the loss of function of *PKD1* or *PKD2*, cause the dysregulation of many pathways such as cAMP, calcium and mTOR signaling cascades, which promote cell proliferation and apoptosis. The hallmark of the disease is the spontaneous generation and constant growth of kidney cysts which gradually expand to demolish the normal renal parenchyma, leading to end stage renal disease (ESRD).

We have recently shown that selective activation of the extracellular calcium-sensing receptor (CaSR) in human conditionally immortalized proximal tubular epithelial cells (ciPTEC), silenced for PKD1 or generated from an ADPKD1 patient, increases cytosolic calcium, and reduces intracellular cAMP and mTOR activity, reversing the principal dysregulations considered the most proximal events in ADPKD pathogenesis, making CaSR a possible candidate as therapeutic target (Di Mise et al., 2018). On the other hand, reduced intracellular calcium observed in ADPKD can affect mitochondrial function and ATP production with consequence on glucose metabolism (Rowe et al., 2013), as showed in recent studies which reported defective glucose metabolism in ADPKD-affected cells and tissues (Rowe et al., 2013; Menezes et al., 2016). They demonstrated that PC1 lacking cells consume high levels of glucose, preferentially using it in aerobic glycolysis for their energy production (Rowe et al., 2013). Indeed, mitochondrial abnormality exists from an early phase of ADPKD, underlining a relationship between mitochondria and renal polycystic diseases (Li et al., 2012; Rowe et al., 2013; Magistroni and Boletta, 2017; Padovano et al., 2017). In line, an association between mitochondria abnormalities and cystogenesis has been reported in cyst-lining cells in ADPKD model mice and in rats (Ishimoto et al., 2017). Recent clinical studies indicate that oxidative stress is already present in the early stages of ADPKD, even when renal function is preserved (Menon et al., 2011; Klawitter et al., 2014), showing that mitochondria, representing the primary reactive oxygen species source, play a functional role in cyst formation. However, the precise pathophysiological role of mitochondria in ADPKD remains elusive.

We now report that CaSR activation in ciPTEC stably knocked down for PC1 restores the decreased mitochondrial calcium levels and fully reverse the deficient ATP production.

## Materials and methods

### Materials

All chemicals were purchased from Sigma (Sigma-Aldrich, Milan, Italy). NPS-R568 was kindly gifted by Amgen (Amgen Dompé S.p.a., Milan, Italy). Media for cell culture were from Lonza (Lonza s.r.l., Milan, Italy). pcDNA-4mtD3cpv was a gift from Amy Palmer & Roger Tsien (Addgene plasmid #36324).

### Antibodies

Monoclonal CaSR antibody recognizing amino-acid 15–29 at the extracellular N-terminus was from Sigma-Aldrich, Milan, Italy. Secondary goat anti-mouse IgG biotin antibodies were purchased from Sigma-Aldrich, Milan, Italy. Streptavidin-488 conjugate were from Alexa Fluor (Molecular Probes, Eugene, Oregon, USA).

### Generation of ciPTEC knocked down for polycystin-1

ciPTEC were generated as described by Wilmer et al. (2010). Briefly, primary cells were cultured by collecting mid-stream urine within 5 h after collection. Urine sediment was resuspended in DMEM Ham's F12 medium supplemented with 10% fetal bovine serum (FBS), 100 IU/ml penicillin, 100 μg/ml streptomycin, ITS (5 μg/ml insulin, 5 μg/ml transferrin and 5 ng/ml selenium), 36 ng/ml hydrocortisone, 10 ng/ml epidermal growth factor (EGF), and 40 pg/ml triiodothyronine. The suspension was placed at 37°C in a 5% CO_2_ incubator.

Primary cells were immortalized as previously described (Wilmer et al., 2010). Cells were infected with SV40T and hTERT vectors, containing, respectively, geneticin (G418) and hygromycin resistance (O'hare et al., 2001; Satchell et al., 2006). Subconfluent cell layers were then grown at 33°C and selected by using G418 (400 μg/ml) and hygromycin B (25 μg/ml) for 10 days. Stable knocked down ciPTEC for polycystin-1 (ciPTEC-PC1KD) were obtained transducing a cloned ciPTEC line (ciPTECwt) of a healthy individual (34.8) by adding lentiviral vectors encoding miR-shRNA directed against polycystin-1, cloned in tandem (pCHMWS Bsd 2xmiRNA PKD1), to the culture medium (Mekahli et al., 2012). Transduced cells were selected using 10 g/ml blasticidin. Experiments were performed prior cellular maturation for 11 days at 37°C. The reduced expression of PC1 was showed by Mekahli and coworkers which biochemically characterized these cell lines (Mekahli et al., 2012).

### Measurement of mitochondrial ATP production rate in ciPTEC

The rate of mitochondrial ATP production was determined in ciPTEC permeabilized with digitonin (0.01%) essentially as already reported (Valenti et al., 2016). Briefly, cells (0.3–0.4 mg protein) were incubated at 37°C in 2 ml of respiratory medium consisting of 210 mM mannitol, 70 mM sucrose, 3 mM MgCl_2_, 20 mM Tris/HCl, 5 mM KH_2_PO_4_/K_2_HPO_4_, (pH 7.4) plus 5 mg/ml BSA, in the presence of the coupled enzyme system revealing ATP, indicated as ATP detecting system (ATP-ds), containing the substrates glucose (2.5 mM) and NADP^+^ (0.25 mM) and the coupled enzymes hexokinase (HK, 3 e.u.) and glucose 6-phosphate dehydrogenase (G6P-DH, 2 e.u.). The assay has been performed in the presence of 0.01 mM diadenosine pentaphosphate (Ap5A) to selectively inhibit the mitochondrial adenylate kinase. The measure has been carried out by adding as energy sources, either glutamate (GLU) plus malate (MAL) (5 mM each) or succinate (SUCC, 5 mM) plus rotenone (ROT, 3 μM), or ascorbate (ASC, 0.5 mM) plus N,N,N′,N′- tetramethyl-p-phenylenediamine (TMPD, 0.25 mM). After 5 min of incubation with digitonin (0.01% w/v), ADP (0.5 mM) was added to start the reaction, and the reduction of NADP^+^ in the extra-cellular phase was monitored as an increase in absorbance at 340 nm. As a control, the ATP synthase inhibitor oligomycin (OLIGO, 5 μg/10 μl) was added during the reaction to ascertain the inhibition of the mitochondrial ATP production. Attention was reserved to use excess HK/G6P-DH coupled enzymes to guarantee a non-limiting ADP-restoring system for the assay of ATP production.

### Measurement of ATP levels in ciPTEC

ciPTEC were detached from plate, washed with PBS and cellular ATP was extracted by using the boiling water procedure, as described in Yang et al. (2002). The amount of intracellular ATP was determined enzymatically in the extracts, as described in Valenti et al. (2010). Cells were left under basal condition or stimulated with NPS-R568 (10 μM for 60 min at 37°C) in Ringer's solution containing 120 mM NaCl, 4 mM KCl, 15 mM NaHCO_3_, 1 mM MgCl_2_, 15 mM Hepes, 0.5 mM NaH_2_PO_4_, 10 mM Glucose, 2 mM CaCl_2_, 0.5 mM Na_2_HPO_4_, 0.4 mM MgSO_4_, pH 7.4 (modified by Mekahli et al., 2012; Miyakawa et al., 2013).

### Fluorescence resonance energy transfer (FRET) measurements

To evaluate mitochondrial calcium content, fluorescence resonance energy transfer (FRET) experiments were performed as described (Di Mise et al., 2015, 2018). Briefly, ciPTEC were seeded onto 20-mm glass coverslips at 37°C for 11 days and were transiently transfected with a plasmid encoding a mitochondrially targeted cameleon containing a mutant calmodulin (mCaM) sequence cloned between CFP and circularly permuted Venus (cpV) (Palmer et al., 2006). Experiments were performed 24 h post-transfection. Cells were left under basal condition or stimulated with NPS-R568 (10 μM for 60 min at 37°C) in Ringer's solution, described above, containing 2 mM CaCl_2_.

FRET measurements were carried out using MetaMorph software (Molecular Devices, MDS Analytical Technologies, Toronto, Canada). CFP and YFP were excited at 436 and 500 nm, respectively; fluorescence emitted was measured at 480/40 nm for CFP and 535/30 nm for YFP and FRET. Corrected normalized FRET values were determined as already described (Rodighiero et al., 2008; Tamma et al., 2013, 2017; Russo et al., 2017). Each image was corrected for CFP cross-talk and YFP cross-excitation. Therefore, netFRET = [IFRETbg – ICFPbg·K1 – IYFPbg·(K2-αK1)]/(1-δK1) where IFRETbg, ICFPbg, and IYFPbg are the background-corrected pixel gray values measured in the FRET, CFP, and YFP windows, respectively; K1, K2, α, and δ are calculated to evaluate the crosstalk between donor and acceptor. The integrated fluorescence density values of the images from each cell were analyzed using MetaMorph and Microsoft Excel software.

### Immunofluorescence microscopy

CaSR immunofluorescence localization in polarized ciPTEC was performed as previously described (Jansen et al., 2014; Di Mise et al., 2015). ciPTEC were cultured on polyester Transwell inserts, left 11 days at 37°C for maturation, and then fixed using 2% (w/v) paraformaldehyde in HBSS with addition of 2% (w/v) sucrose for 5 min and permeabilized in 0.3% (v/v) triton X-100 in HBSS for 10 min.

Cells were incubated with antibodies diluted in block solution containing 2% (w/v) bovine serum albumin (BSA) and 0.1% (v/v) tween-20 in HBSS against the calcium-sensing receptor (CaSR) at 4°C overnight. Following treatment with secondary rabbit-anti-mouse-biotin antibodies followed by Streptavidin-488, samples were mounted on glass slides with Mowiol. Images were obtained with a confocal microscope Leica TCS SP2 (Leica Microsystems, Heerbrugg, Switzerland).

### Statistical analysis

Statistical analysis was performed using One-way ANOVA followed by Newman–Keuls multiple comparisons test or Two-way ANOVA followed by Turkey's multiple comparisons test or *t-*test. All values are expressed as means ± SEM. A difference of *P* < 0.05 was considered statistically significant.

## Results and discussion

### Impaired bioenergetics status in ciPTEC silenced for PC1

Emerging evidence of inherent metabolic reprogramming in *PKD1* knockout cells suggests that the PC1-PC2 complex regulates cellular metabolism (Menezes et al., 2016; Padovano et al., 2017). The exact nature of the metabolic alterations remains controversial, with some groups reporting enhanced glycolysis, reminiscent of the Warburg phenomenon (Rowe et al., 2013; Padovano et al., 2017), and others reporting no evidence for a glycolytic switch (Menezes et al., 2016; Warner et al., 2016), proposing instead fatty acid oxidation impairment (Menezes et al., 2016; Hajarnis et al., 2017).

Lin and collaborators (Lin et al., 2018) suggest a direct link between PKD proteins and control of mitochondrial activity, showing that *PKD1* knockout cells have different metabolic fluxes with likely altered oxidoreductase activity, consistent with changes in NAD^+^/NADH ratio. Furthermore, their data document altered mitochondrial membrane potential and abnormal mitochondrial networks in *PKD1* knockout cell lines. Morphological abnormalities of mitochondria have also been found in human ADPKD cyst-derived cells with heterozygous and homozygous *PKD1* mutation, indicated to facilitate cyst formation in ADPKD (Ishimoto et al., 2017).

**Figure 1:**
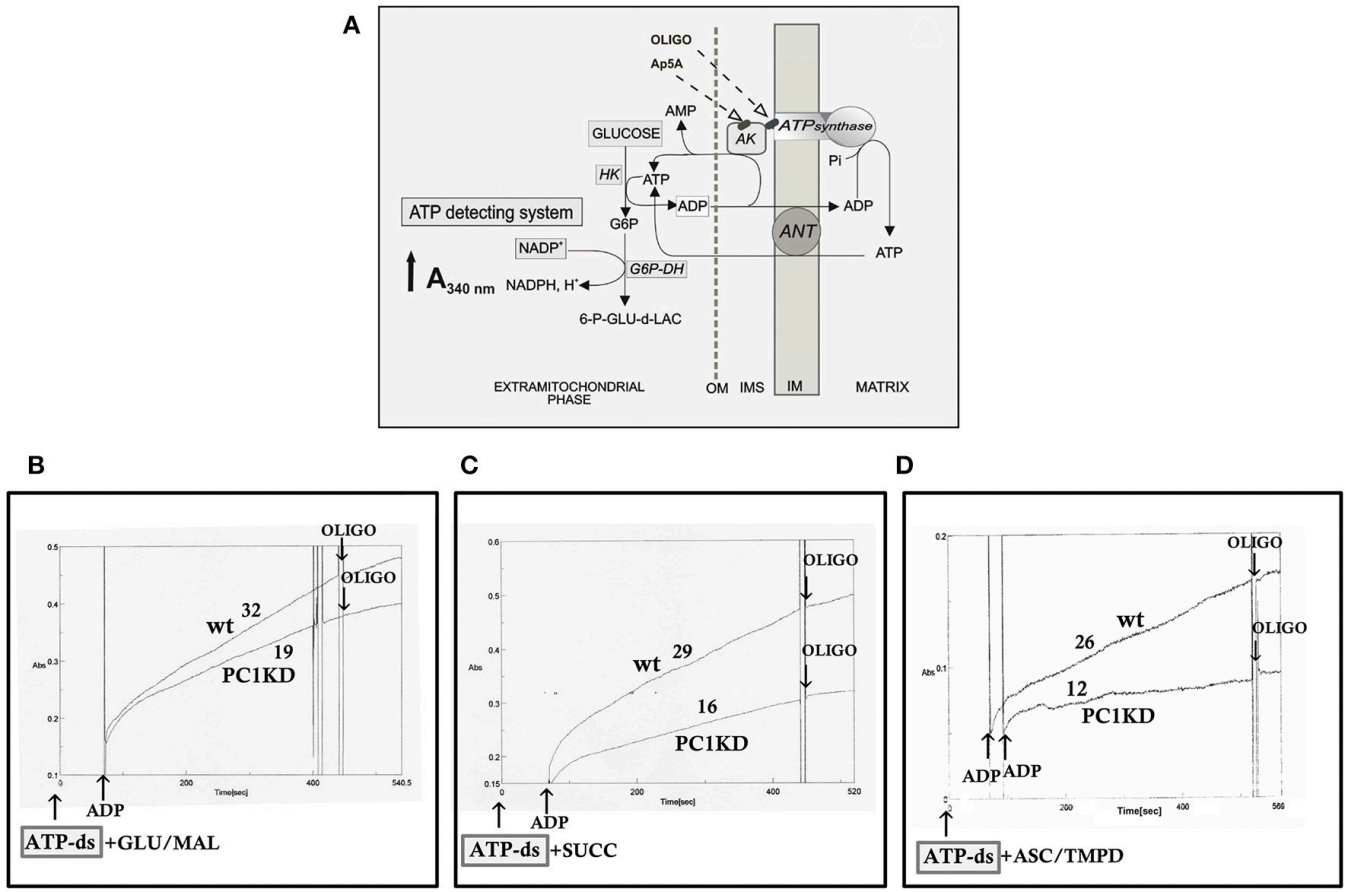
Impaired mitochondrial ATP synthesis in human ciPTEC-PC1KD. **(A)** Schematic representation of the mitochondrial ATP synthesis revealed by ATP detecting system (ATP-ds). ANT, adenine nucleotide translocator; AK, adenylate kinase; HK, hexokinase; G6P-DH, glucose 6-phosphate dehydrogenase; 6P-GLUC- δ-LAC, 6 phosphogluconate-δ-lactone; IMS, intermembrane space; MIM, mitochondrial inner membrane; OM, outer membrane. **(B–D)** Representative spectrophotometric traces of mitochondrial ATP production in human ciPTEC. Either human ciPTEC stably silenced for polycystin-1 (ciPTEC-PC1KD) or wild type clone (ciPTECwt) (0.3 mg) were permeabilized with 0.01% digitonin for 5 min and incubated at 37°C in 2 ml of respiration medium in the presence of ATP-ds plus the indicated respiratory substrates. **(B)** Glutamate plus malate (GLU/MAL, 5 mM each), **(C)** 5 mM succinate (SUCC) plus 3 μM rotenone, **(D)** 5 mM ascorbate plus 0.5 mM TMPD (ASC/TMPD). Where indicated, ADP (0.5 mM) was added. At the arrows, the ATP synthase inhibitor oligomycin (OLIGO, 5 μg/10 μl) was added in course of reaction. The numbers along curves represent the rates of the increase in absorbance at 340 nm, measured as tangents to the initial slopes and expressed as nmol of NADPH formed/min per mg of protein.

On this basis, to investigate whether the bioenergetics status is affected in ciPTEC stably silenced for PC1, we first evaluated the mitochondrial ATP synthesis in ciPTEC-PC1KD cells compared with wild type clone, by monitoring in *continuous* the ATP produced by mitochondria *in situ* and flowed outside cells, under conditions where oxidative phosphorylation (OXPHOS) can take place (Valenti et al., 2010). ciPTEC-PC1KD or ciPTECwt, permeabilized with digitonin (0.01%), were incubated with the ATP detecting system (ATP-ds) and the efflux of ATP in the extracellular phase occurring after the addition of ADP was monitored as an increase in absorbance at 340 nm due to NADPH synthesis, and evaluated as a measure of mitochondrial ATP production (Figure 1A).

**Figure 2:**
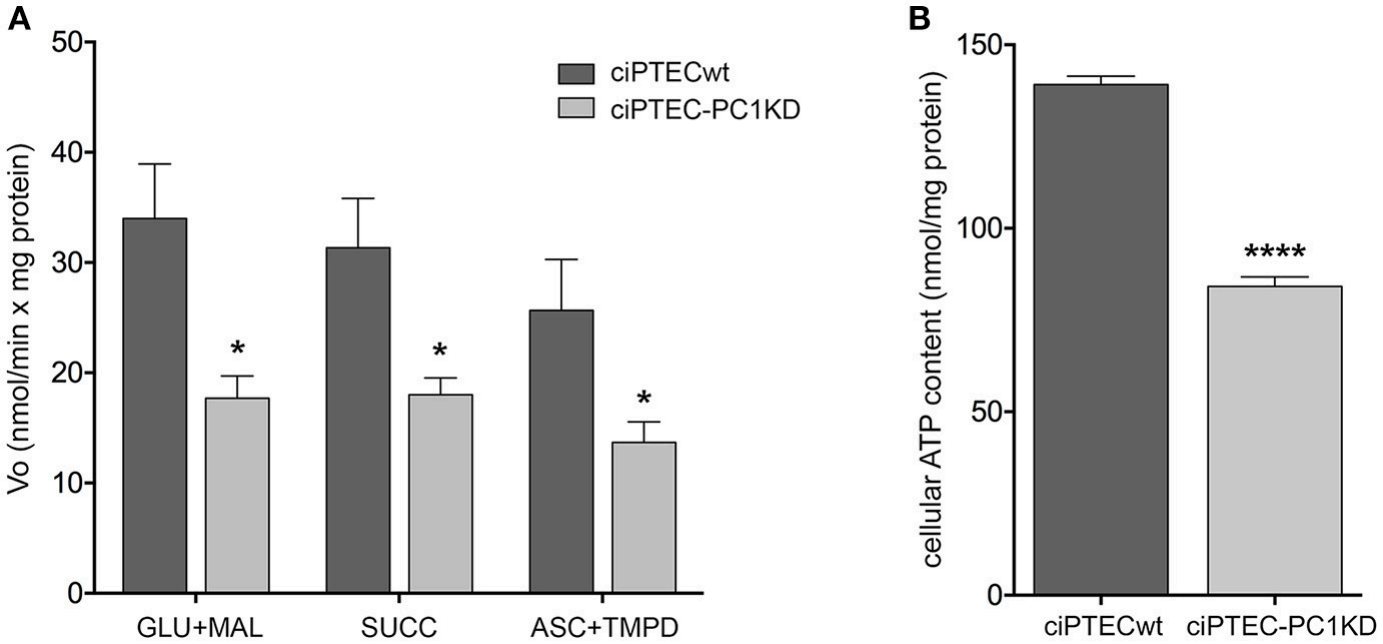
Impaired bioenergetics status in human ciPTEC-PC1KD. **(A)** The rate of mitochondrial ATP production was measured in human ciPTEC stably silenced for polycystin-1 (ciPTEC-PC1KD) vs. wild type clone (ciPTECwt), in the presence of the respiratory substrates GLU plus MAL (GLU/MAL, 5 mM each) or 5 mM SUCC plus 3 μM rotenone, or 5 mM ASC plus 0.5 mM TMPD (ASC/TMPD), as described in the legend of Figure 1. Values are means ± SEM (*P < 0.01 vs. ciPTECwt) obtained from three independent experiments and expressed as nmol NADPH/min per mg protein. **(B)** Cellular ATP levels were measured in ciPTEC-PC1KD vs. matched ciPTECwt, as described under Methods. Each histogram is representative of four independent experiments. Data are expressed as means ± SEM. Significant differences with respect to wt were calculated by Student's *t-*test (****P < 0.0001).

To give a more complete evidence on the efficiency of the OXPHOS system, and disclose the distinctive contribution of each mitochondrial respiratory chain complex composing the OXPHOS apparatus, the mitochondrial ATP synthesis was measured by supplying as energy sources the respiratory substrates of complex I glutamate plus malate (GLU+MAL), complex II succinate (SUCC) or complex IV ascorbate plus TMPD (ASC+TMPD). As shown by the representative spectrophotometric traces (Figures 1B–D) and by the histograms reporting the statistical analyses of data (Figure 2A), a drastic reduction in the rate of mitochondrial ATP synthesis was found, commonly shared when supplied ciPTEC-PC1KD with GLU+MAL (estimated as 41 ± 13% vs. ciPTECwt, *P* < 0.01) or SUCC (estimated as 39 ± 10% vs. ciPTECwt, *P* < 0.01) or ASC+TMPD (estimated as 56 ± 11% vs. ciPTECwt, *P* < 0.01), thus indicating a multilevel inhibition of the mitochondrial ATP production by OXPHOS in ciPTEC-PC1KD.

To assess whether the compromised mitochondrial energy efficiency found in ciPTEC-PC1KD could impact the cell energy status, the cellular ATP pool was measured (Figure 2B). ATP level was found significantly reduced in ciPTEC-PC1KD (estimated as 38 ± 4%, *P* < 0.0001) with respect to wt, suggesting that the deficit in ATP production by mitochondria reflects and mirror the overall cellular energy level in ciPTEC-PC1KD.

These data indicate an impairment in mitochondrial OXPHOS associated with a great shortage in cell energy status in *PKD1* silenced cells. How the lack of *PKD1* causes such a mitochondrial dysfunction in ciPTEC it is not yet clear. Both transcriptional and/or post-translational mechanisms regulating OXPHOS process may be involved. Among post-translational regulation of OXPHOS, calcium signaling between cytosol and matrix primarily coordinates the crosstalk of mitochondria within the cell, establishing the balance between the energy requests of the cell and the energy production by mitochondrial OXPHOS (Glancy and Balaban, 2012).

### Reduced mitochondrial calcium content in ciPTEC-PC1KD

It has been shown that cultured epithelial cells derived from human ADPKD cysts exhibit a reduction in steady state cytosolic calcium levels with respect to normal human kidney cells (Yamaguchi et al., 2006). Moreover, PC complex interacts with other calcium channels expressed in the ER, displaying a pivotal role in the prevention of intracellular stores depletion, especially of the ER itself (Anyatonwu et al., 2007; Weber et al., 2008; Santoso et al., 2011). Recently, we demonstrated that, compared to ciPTECwt, ciPTEC-PC1KD have significantly lower calcium concentration both in the cytosol and in the ER, highlighting that the loss-of-function mutation in *PKD1* is strictly connected to the dysregulation of the two intracellular calcium bulks, likely secondary to the PC complex disruption and dysfunction (Di Mise et al., 2018).

The ER is known to be functionally associated to mitochondria with a rapid transport of calcium across their membranes and its accumulation in the mitochondrial matrix, where several calcium effectors are located (Tarasov et al., 2012). The inositol 1,4,5-trisphosphate receptors (IP3Rs) are the main drivers of the ER-to-mitochondria Ca^2+^ shuttle supporting cellular bioenergetics (Cárdenas et al., 2010). Mitochondrial calcium uptake by IP3R-released Ca^2+^ is fundamentally required to maintain adequate mitochondrial NADH production to sustain OXPHOS in resting cells (Cárdenas et al., 2010). IP3R activity is linked to regulation of cellular bioenergetics through calcium-dependent activation of mitochondrial ATP synthesis, by triggering key matrix enzymes, including pyruvate dehydrogenase, α-oxoglutarate dehydrogenase, isocitrate dehydrogenase, as well as downstream elements of OXPHOS, comprising F1/Fo ATP synthase and the cytochrome chain (Glancy and Balaban, 2012).

**Figure 3:**
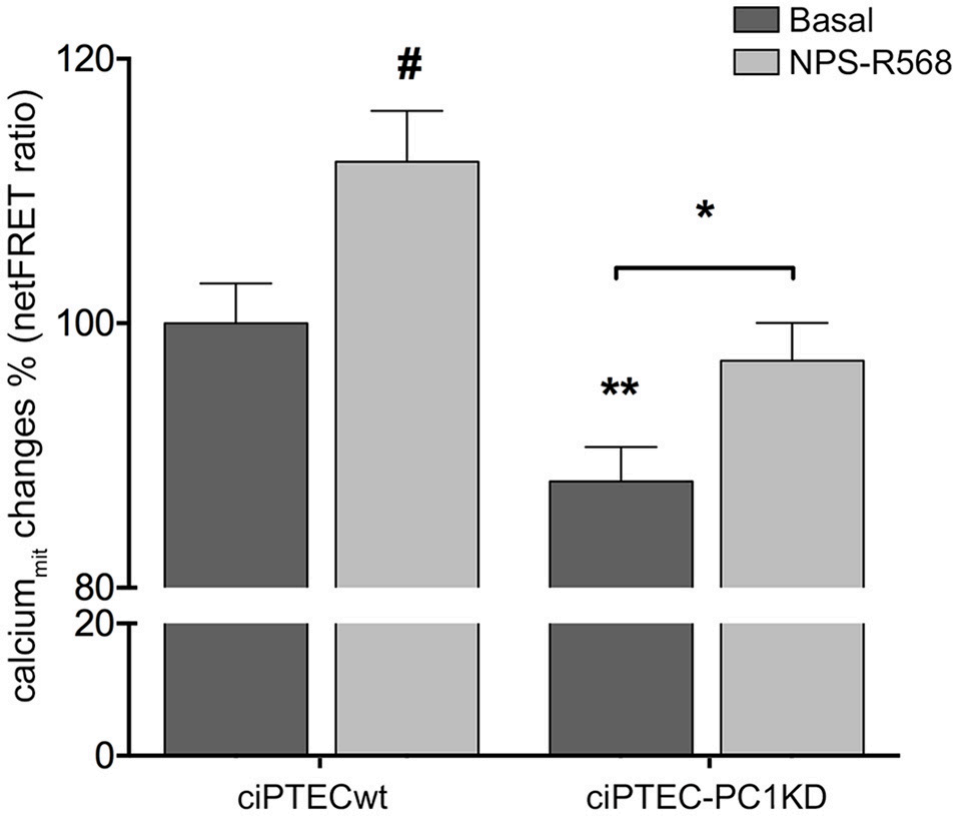
Reduced mitochondrial calcium levels in human ciPTEC-PC1KD: effect of CaSR positive allosteric modulator NPS-R568. Evaluation of mitochondrial Ca^2+^ with mitochondria-targeted cameleon (4mtD3cpV) FRET probe. Histogram compares changes in normalized FRET (netFRET) ratio between ciPTECwt and ciPTEC-PC1KD, at basal conditions or treated with NPS-R568. At rest, ciPTEC-PC1KD presented a significant lower mitochondrial calcium content with respect to ciPTECwt (**P < 0.01). CaSR stimulation with NPS-R568 10 μM induced a significant increase in calcium levels in ciPTEC-PC1KD, restoring levels close to ciPTECwt at basal conditions. All data were analyzed by One-way ANOVA followed by Newman–Keuls multiple comparisons test and expressed as means ± SEM (*P < 0.01 ciPTEC-PC1KD+NPS-R568 vs. ciPTEC-PC1KD Basal; ^#^*P* < 0.001 ciPTECwt+NPS-R568 vs. ciPTECwt Basal).

In line with these findings, the resting mitochondrial calcium content in ciPTEC-PC1KD resulted significantly reduced with respect to wt cells (ciPTEC-PC1KD = 88.05 ± 2.6%, *n* = 140, vs. ciPTECwt = 100%, *n* = 192; *P* < 0.01; Figure 3). These data further confirm the functional interaction between ER and mitochondria that are in close apposition, allowing calcium transfer between these organelles and supporting the hypothesis that the PCs are involved in the regulation of mitochondrial calcium levels, as previously suggested (Patergnani et al., 2011; Padovano et al., 2017). Indeed, in line with this hypothesis, PCs have been shown to co-fractionate with mitochondria-associated ER membranes (MAMs) (Padovano et al., 2017).

### Activation of CaSR by NPS-R568 results in full recovery of mitochondrial calcium levels and restoring of cell energy deficit in ciPTEC-PC1KD

**Figure 4:**
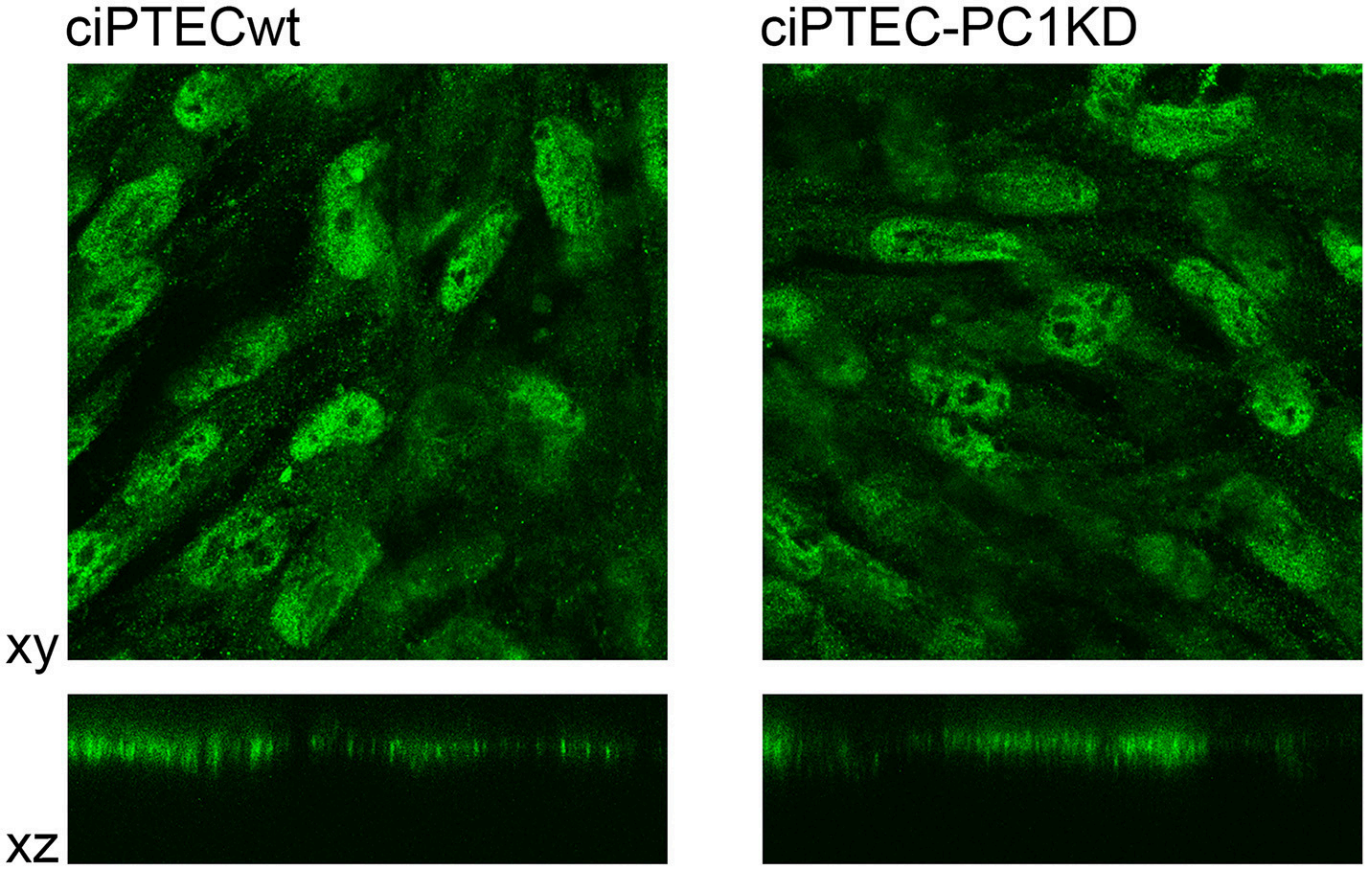
Immunofluorescence localization of endogenous CaSR in human ciPTEC cells. In polarized ciPTEC, CaSR shows a predominant apical plasma membrane localization as occurs in native proximal tubule epithelial cells.

ciPTEC stably silenced for PC1 as well as wild type clone, express endogenous CaSR mainly localized to the apical plasma membrane as it occurs in native renal proximal tubule epithelial cells (Figure 4) (Riccardi and Valenti, 2016; Di Mise et al., 2018). CaSR is coupled to three main groups of heterotrimeric G proteins, G_q/11_, G_i_, and G_12/13._ Recently we have shown that CaSR expressed in ciPTEC interacts with G_q_ as a downstream effector (Di Mise et al., 2015). As known, when CaSR is activated, it induces cytosolic calcium increase via G_q_ activation which stimulates PLC with subsequent IP3 and diacylglycerol production. IP3 binds to high affinity receptors on the ER (IP3Rs), causing the release of calcium into the cytoplasm.

On this basis, we evaluated the effect of CaSR stimulation with NPS-R568 on mitochondrial calcium levels. NPS-R568 treatment resulted in a significant increase in ciPTEC-PC1KD mitochondrial calcium (ciPTEC-PC1KD+NPS-R568 = 97.17 ± 2.87%, *n* = 200), which raised close to the basal levels observed in ciPTECwt (Figure 3). This result suggests that the increase in mitochondrial calcium is a consequence of the CaSR activation induced signaling which leads to IP3 production and calcium influx from ER to the mitochondrion through IP3Rs (Cárdenas et al., 2010). Moreover, the increase of cytosolic Ca^2+^ elicited by CaSR activation also contributes to the increment of mitochondrial Ca^2+^ content via the mitochondrial calcium uniporter (MCU) (Marchi and Pinton, 2014).

As known, mitochondrial calcium homeostasis and uptake has a pivotal role in the regulation of mitochondrial ATP generation as well as cytosolic NAD^+^/NADH metabolism, thus sustaining the energy requirements of the cell (Raffaello et al., 2016; Arduino and Perocchi, 2018).

**Figure 5:**
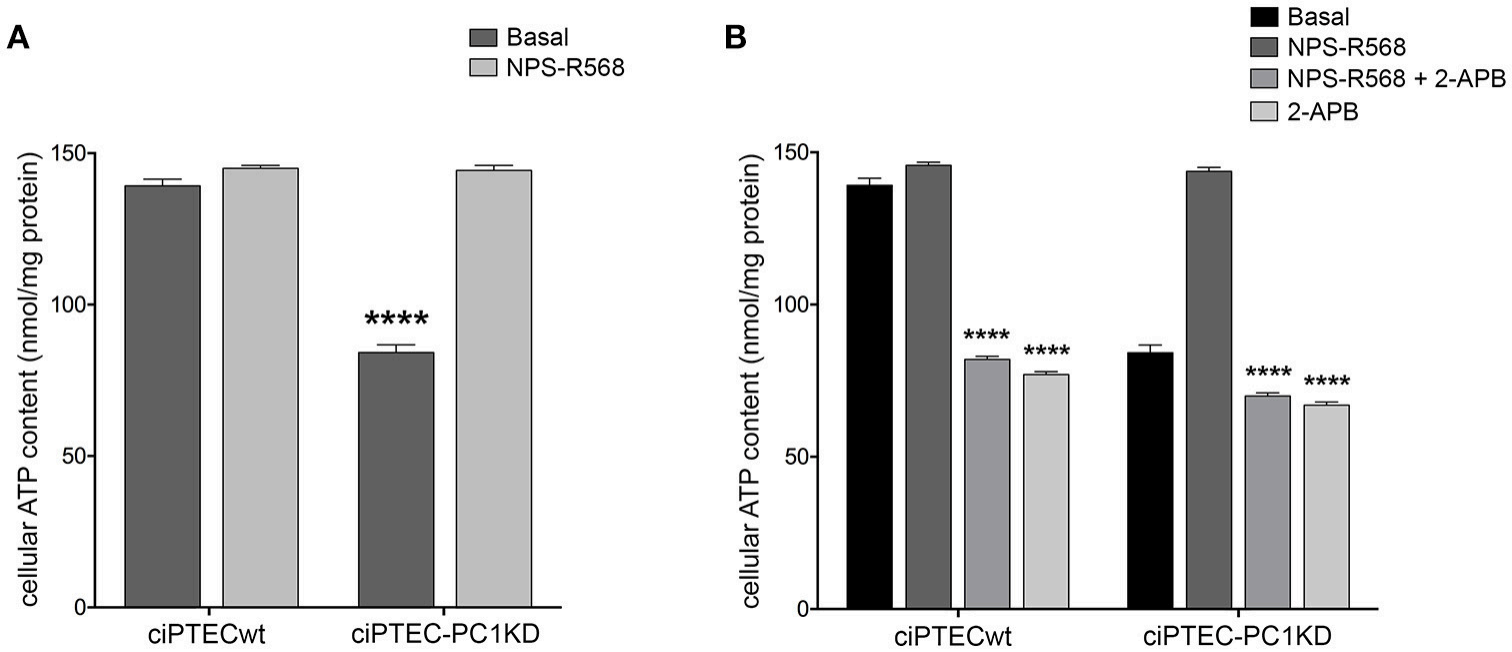
NPS-R568 treatment results in a complete recovery of cell energy status in human ciPTEC-PC1KD. The ATP level in human ciPTEC was measured in three independent experiments for each experimental group and expressed as nmol ATP/mg protein. **(A)** CaSR stimulation elicited by NPS-R568 induced a significant increase in cellular ATP content in ciPTEC-PC1KD, restoring levels comparable to ciPTECwt at basal conditions. Data were analyzed by One-way ANOVA followed by Newman–Keuls multiple comparisons test and are expressed as means ± SEM (****P < 0.0001 vs. ciPTECwt Basal or ciPTEC-PKD1+NPS-R568). **(B)** IP3R inhibition with 2-APB in ciPTEC-PC1KD prevented cellular ATP levels rescue induced by CaSR activation. Data were analyzed by Two-way ANOVA followed by Turkey's multiple comparisons test and are expressed as means ± SEM (****P < 0.0001 vs. NPS-R568 treatment in each cell line).

Remarkably, ciPTEC treatment with the calcimimetic NPS-R568 resulted in a full recovery of cellular ATP content in ciPTEC-PC1KD (ciPTEC-PC1KD+NPS-R568 = 144.3 ± 1.67 nmol/mg protein), restoring the levels measured in ciPTECwt at basal conditions (ciPTECwt = 139.2 ± 2.27 nmol/mg protein; Figure 5A). To assess the strict involvement of calcium transfer from ER to mitochondria in sustaining cellular bioenergetics thus rescuing energy deficit in PC1 knockdown cells after CaSR activation, ciPTEC were treated with 2-aminoethoxydiphenyl borate (2-APB), a selective IP3R inhibitor (Szatkowski et al., 2010). Furthermore, to abolish the calcium amount permeating mitochondria directly from the cytosol via MCU, we blocked IP3Rs with 2-APB in presence of BAPTA-AM, a cell-permeant highly selective calcium chelator (Gerbino et al., 2009). 2-APB treatment of ciPTEC-PC1KD completely prevented the rescue of bioenergetics deficit induced by NPS-R568, showing values comparable to the intracellular ATP content measured under basal conditions (70.6 ± 0.9 nmol/mg protein; Figure 5B). A similar reduction in cellular ATP was reported in ciPTECwt treated with 2-APB (82.4 ± 0.82 nmol/mg protein; Figure 5B), suggesting a critical role of IP3Rs in driving ER-to-mitochondria Ca^2+^ shuttle supporting cellular bioenergetics. BAPTA-AM *per se* did not affect ATP cellular levels (data not shown).

**Figure 6:**
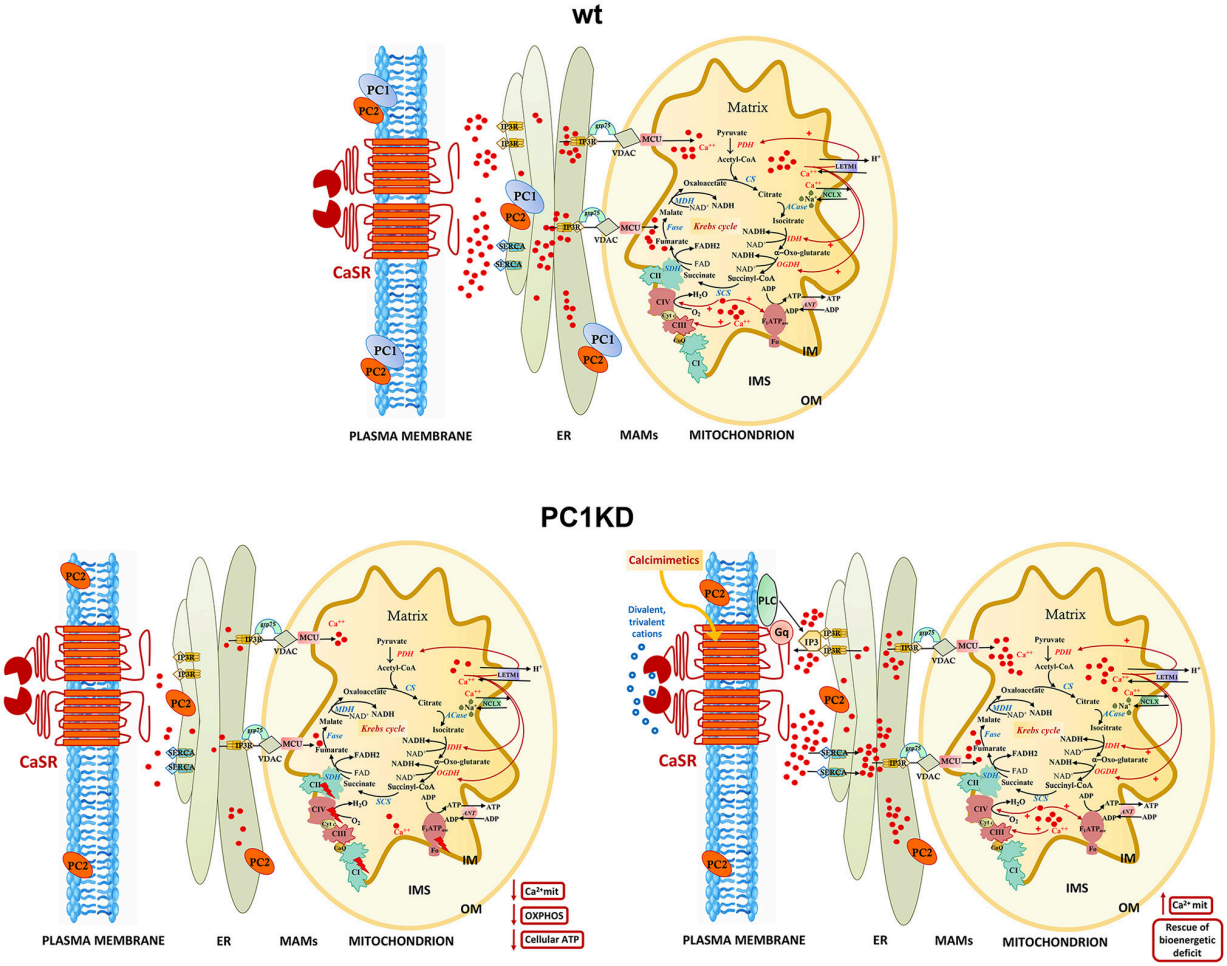
Models of intracellular calcium signaling network and mitochondrial calcium regulation of cellular bioenergetics in wt, PC1KD, and calcimimetic-treated PC1KD cells. Mitochondria are main players in calcium signaling network, skillful in regulating both the extent and the spatial/temporal Ca^2+^ signals. The membrane micro-domains between the ER and mitochondria -named Mitochondria Associated Membranes (MAMs)- are critical for an efficacy inter-organelle crosstalk (Rieusset, 2018). Several proteins, including IP3R, grp75, and VDAC, are involved in Ca^2+^ release from the ER, ensuring an active mitochondrial Ca^2+^ uptake. The mitochondrial Ca^2+^ uniporter (MCU) and the H^+^/Ca^2+^ exchanger (LETM1) mediate the Ca^2+^ uptake from cytosol inside mitochondria; conversely, the mitochondrial Na^+^/Ca^2+^ exchanger (NCLX), catalyzes the mitochondrial Ca^2+^ export. Inside the mitochondrion are schematized the interactions of matrix Ca^2+^ with processes involved in oxidative phosphorylation. The red arrows from Ca^2+^ to the different protein targets indicate either a direct or indirect modulating effect on their enzymatic or transport activities. Under basal conditions, PC1KD cells show lower mitochondrial Ca^2+^ levels with respect to wt, associated to a multilevel inhibition of OXPHOS apparatus, leading to a severe deficit of mitochondrial ATP production and cellular energy status. CaSR stimulation with the calcimimetic NPS-R568 rises intracellular calcium levels triggering the sarco/endoplasmic reticulum Ca^2+^-ATPase (SERCA) which drives Ca^2+^ from the cytosol to the lumen of the ER. CaSR activation prompts phospholipase C (PLC), a phosphodiesterase responsible for the hydrolysis of phosphatidylinositol 4,5-biphosphate to acil-glycerol and inositol 3 phosphate (IP3). The binding of IP3 to its receptor IP3R induces Ca^2+^ release from ER and transfer of Ca^2+^ from ER to mitochondria at the contact sites between ER and mitochondria. CaSR activation by calcimimetic increases mitochondrial Ca^2+^ levels and full restores the bioenergetics deficit in PC1KD. Enzyme, channel, and carrier abbreviations: PDH, pyruvate dehydrogenase; CS, citrate synthase; ACase, aconitase; IDH, isocitrate dehydrogenase; OGDH, oxoglutarate dehydrogenase; SCS, succinyl CoA synthase; SDH, succinate dehydrogenase (component of Complex II); Fase, fumarase; MDH, malate dehydrogenase; VDAC, voltage-dependent anion channel; ANT, adenine nucleotide translocator; IP3R, inositol 3-phosphate receptor. The complexes of oxidative phosphorylation are labeled as roman numerals from I to IV.

These findings point to a crucial role of mitochondrial calcium in regulating the mitochondrial energy status. Indeed, calcium is actively transported inside mitochondria and accumulates in the mitochondrial matrix, where several calcium protein targets are located (Rizzuto et al., 2012) (details in the model in Figure 6). In this context, the existence of high-calcium micro-domains between ER and mitochondria ensures efficient calcium transfer from ER to mitochondria (Rizzuto et al., 1998; Csordás et al., 1999, 2010), which is mediated by a multiprotein complex composed—among others—of the IP3R at ER membrane and of the VDAC at outer mitochondrial membrane (OMM) (Szabadkai et al., 2006). The zones of close contact between the ER and mitochondria, called Mitochondria Associated Membranes (MAMs), are crucial for a correct crosstalk between the two organelles (Csordás et al., 2006). Therefore, MAMs disruption causes the suppression of the IP3-mediated release of calcium from the ER to mitochondria, with a consequent reduction of ATP production (Rowland and Voeltz, 2012).

A model of intracellular calcium signaling network in PC1 deficient cells exposed to calcimimetic is shown in Figure 6.

To summarize, the present contribution provides the first evidence that selective CaSR activation in human ciPTEC stably knocked down for PC1, restores mitochondrial calcium content and fully rescues the bioenergetics dysfunction known to facilitate cysts formation in ADPKD. These data, together with our previous demonstration that CaSR activation in ciPTEC-PC1KD increases cytosolic calcium and decreases cAMP and mTOR activity, indicate that CaSR signaling can reverse the principal dysregulations considered the most proximal events in ADPKD pathogenesis, making CaSR a potential candidate as therapeutic target.

## Author contributions

GV and DV designed research and supervised the project. DV and AD designed, performed, and interpreted experiments. MR, MC, MV, and GT performed research and analyzed data. AD, GV, and DV wrote the paper. All authors commented on the manuscript.

### Conflict of interest statement

The authors declare that the research was conducted in the absence of any commercial or financial relationships that could be construed as a potential conflict of interest.
